# Piezoresistive Theory and Numerical Calculation for Carbon Nanotube Polymer Composite

**DOI:** 10.3390/ma16227090

**Published:** 2023-11-08

**Authors:** Zhengwei Huang, Ying Song, Xiaohua Zhao, Huiming Hou

**Affiliations:** 1 Department of Civil and Environmental Engineering, Shantou University, Shantou 515063, China; 19zwhuang@stu.edu.cn (Z.H.); 12ysong@alumni.stu.edu.cn (Y.S.); xhzhao@stu.edu.cn (X.Z.); 2 Key Laboratory of Structure and Wind Tunnel of Guangdong Higher Education Institutes, Shantou 515063, China

**Keywords:** carbon nanotubes, composites, electrical conductivity, piezoresistivity, finite element

## Abstract

A three-dimensional theory has been established for the piezoresistivity of carbon nanotube (CNT) polymer composites. Based on the Mori–Tanaka method in meso-mechanics theory and considering quantum tunneling effect between CNTs, an approach to calculate equivalent electrical conductivity of composites was proposed. On this basis, a piezoresistive theory, which incorporates the effect of composites’ geometric nonlinearity, was developed for CNT polymer composites. The theory is dependent only on some basic physical parameters of the materials. A finite element formula of the theory for the numerical calculation of piezoresistivity was presented from the analysis of both elastic and electric fields. Numerical simulations demonstrated that the results predicted by the theory were in good agreement with those of the experimental tests. Parameter sensitivity analysis revealed that when both the potential barrier height of the matrix and the initial average separation distance between CNTs increased, the piezoresistivity obviously increased. However, with the increase in aspect ratio and CNT conductivity, the piezoresistivity decreased gradually. A practical engineering application of this theory is also provided.

## 1. Introduction

Carbon nanotubes (CNTs) have extraordinary mechanical and electrical properties and have been widely used as reinforcements for the fabrication of composite materials in recent years [1,2,3,4,5]. Most CNT composites are polymer-based. It is well-known that polymers are poor in terms of electrical conductivity, only about $10^{-16}\;S/m∼10^{-12}\;S/m$ [6], while the conductivity of carbon nanotubes is $1∼10^{6}\;S/m$ [7]. Therefore, carbon nanotubes can not only improve the mechanical properties of the composite, but also increase its electrical conductivity. Experiments have shown that when the volume fraction of carbon nanotubes exceeds a certain value, the electrical conductivity of the composite increases sharply by more than 10 orders of magnitude, and then increases slowly, showing obvious percolation-like properties [7,8]. In the vicinity of the percolation threshold, CNT composites were found to present piezoresistive behaviors, that is, the electrical conductivity of the composites varied regularly with the variation of the strain [9,10,11]. It was further shown by experimental tests that this piezoresistive property depends on two conductivity mechanisms: the conductive network formed by CNTs and the quantum tunneling effect between adjacent CNTs [7,12,13,14,15]. Due to the piezoresistive properties, CNT composites have various potential applications, such as in smart structures and in the construction of advanced sensors [16]. Therefore, it is of significance to study the piezoresistive property of CNT composites.

A few experiments have been conducted to test the piezoresistivity of CNT composites. Most of them often display two different types of piezoresistive behaviors: (1) piezoresistivity increases linearly when the strain is below a critical value, and (2) beyond the critical strain, piezoresistivity increases exponentially, and the quantum tunneling effect plays a major role [17,18,19]. Since the quantum tunneling conductivity between CNTs varies exponentially with the distance between CNTs, a small variation in composite deformation may lead to a significant change in the conductivity of the composites. More recently, some research in the literature has been devoted to establishing both numerical and analytical models to predict the piezoresistivity of CNT composites, which may provide useful tools for the design of highly sensitive resistance-type strain sensors made with various CNT composites. Alamusi [20] proposed a numerical model to predict the piezoresistive behaviors of CNT composites, where both the conductive network formed by CNTs and the quantum tunneling effect between adjacent CNTs were considered. Wang [15] developed a simple analytical model to predict the piezoresistivity of CNT composites using micromechanics. However, both Alamusi and Wang’s models were one-dimensional and linear, and therefore unable to predict the nonlinear variation trend of piezoresistivity. In order to simulate the strong nonlinearity and asymmetry of piezoresistivity, Gong [21] introduced CNTs’ piezoresistivity (effect of CNT deformation) into his model, believing that CNT deformation is a dominant mechanism for the nonlinearity and asymmetry of piezoresistivity of CNT composites. Hu [22] proposed a comprehensive multi-scale resistor network for the prediction of piezoresistivity numerically, in which three mechanisms were involved: (a) a change of conductive network, (b) the quantum tunneling effect, and (c) CNTs’ piezoresistivity. This study shows that the contribution from CNTs’ piezoresistivity is quite small and can be neglected. Nevertheless, this is inconsistent with Gong’s model.

In fact, experiments [17,18,19] showed that it is only when the critical strain of composites is exceeded that the piezoresistivity will increase non-linearly. Generally, the critical strain is so large that the small deformation hypothesis no longer applies. The significant deformation of composites changes the conductivity network, as well as the quantum tunneling distance between CNTs, leading to the nonlinear variation of piezoresistivity. Therefore, in order to reflect the nonlinear variation of piezoresistivity, it is necessary to consider the effect of geometrical nonlinearity (large deformation) on the piezoresistivity of composites. Unfortunately, there are few theoretical or numerical studies involving geometrical nonlinearity. On the other hand, the models mentioned above only predict one-dimensional piezoresistivity, that is, the piezoresistive effect due to uniaxial tension or compression. In fact, composites are usually subjected to a three-dimensional stress state. Thus, the effect of a complex stress state should be included.

In this study, the piezoresistivity of CNT polymer composites is analytically investigated. Based on the theory of meso-mechanics, a new method to calculate the equivalent conductivity of CNT polymer composites is proposed, which includes the mechanisms of both the quantum tunneling effect and CNTs’ conductive network. On this basis, a three-dimensional theory to predict the piezoresistivity of CNT polymer composites under a complex stress state is established, and a numerical approach using the finite element method is developed. For the theory, the effects of geometrical nonlinearity on CNTs conductivity network and quantum tunneling electrical conductivity are considered. With this theory, both the electrical conductivity and piezoresistivity of CNT composites can be calculated by the use of some basic physical parameters of CNTs and polymers.

Notation: Compact tensor and matrix notation are used in this paper. As general rules, scalars are denoted by italic light-face Greek or Latin letters (e.g., π or f); for vectors, second- and fourth-order tensors are signified by italic boldface characters (e.g., σ or ε); and the matrix is denoted by characters within [ ] (e.g., [σ]).

## 2. Piezoresistivity of CNT Composites Based on Deformation

It is assumed that CNT composites are elastic. The governing equations of displacement u, strain ε, and stress σ are

(1a) ∇⋅σ+f=0              

(1b) $$\varepsilon =\frac{1}{2}\left[\left(\nabla ⊗u\right)+{\left(\nabla ⊗u\right)}^{T}+\left(\nabla ⊗u\right)\cdot {\left(\nabla ⊗u\right)}^{T}\right]\;$$

(1c) σ=D:ε               

where f is the body force and D is the elasticity tensors. Note that in Equation (1b), the geometrical nonlinearity term $\left(\nabla ⊗u\right)\cdot {\left(\nabla ⊗u\right)}^{T}$ is involved.

Boundary conditions:

(2) $$\mathrm{stress}\;\mathrm{boundary}\;\mathrm{conditions}\;\;\;\;\;\;\;\;\;\;\;\;\;\;\;\;\;\;n\cdot \sigma =\bar{T}$$

(3) $$\mathrm{displacement}\;\mathrm{boundary}\;\mathrm{conditions}\;\;\;\;\;\;\;\;\;\;u=\bar{u}$$

where $\bar{T}$ and $\bar{u}$ are boundary tractions and displacements, respectively; n is the outer unit normal vector of boundary.

The potential energy functional of an elastic body is:

(4) $$\pi^{m}=\int_{V}\frac{1}{2}\varepsilon^{T}:D:\varepsilon dv-\int_{V}f\cdot udv-\int_{S}\bar{T}\cdot uds$$

Equation (4) can be rewritten as follows if the elastic body is discretized into a mesh of finite elements:

(5) $$\pi^{m}=\sum_{e}\left(\int_{V^{e}}\frac{1}{2}\varepsilon^{eT}:D:\varepsilon dv-\int_{V}f\cdot u^{e}dv-\int_{S}\bar{T}\cdot u^{e}ds\right)$$

The superscript ‘e’ represents the element, and the superscript ‘T’ denotes transpose.

To facilitate the finite element analysis, matrix formulation was adopted as follows. In this paper, the iso-parametric element of a 20-node hexahedron was used for the discretization. For a particular element, displacements can be represented as:

(6) $$\left[u^{e}\right](x,y,z)=\left[N\right]\left[a^{e}\right]$$

where $\left[u^{e}\right]$ is element displacement vector, $\left[N\right]$ is the shape function matrix, and $\left[a^{e}\right]$ is the column vector of nodal displacements for a particular element. Substitution of Equation (6) into (1b) yields

(7) $$\left[\varepsilon \right]=\left[\varepsilon_{L}\right]+\left[\varepsilon_{NL}\right]$$

where $\left[\varepsilon_{L}\right]=\left[B_{L}\right]\left[a^{e}\right]$ [23]; $\left[\varepsilon_{NL}\right]$ is a nonlinear term, and $\left[\varepsilon_{NL}\right]=\left[B_{NL}\right]\left[a^{e}\right]$.

(8) $$\left[B_{NL}\right]=\left[\left[A_{1}\right]\left[H_{1}\right]\;\left[A_{2}\right]\left[H_{2}\right]\;\cdots \;\left[A_{20}\right]\left[H_{20}\right]\right]$$

(9) $$\left[A_{i}\right]=\frac{1}{2}\left[\begin{array}{ccc} {\left[\lambda_{ix_{1}}\right]}^{T} & 0 & 0\\ 0 & {\left[\lambda_{ix_{2}}\right]}^{T} & 0\\ 0 & 0 & {\left[\lambda_{ix_{3}}\right]}^{T}\\ {\left[\lambda_{ix_{2}}\right]}^{T} & {\left[\lambda_{ix_{1}}\right]}^{T} & 0\\ 0 & {\left[\lambda_{ix_{3}}\right]}^{T} & {\left[\lambda_{ix_{2}}\right]}^{T}\\ {\left[\lambda_{ix_{3}}\right]}^{T} & 0 & {\left[\lambda_{ix_{1}}\right]}^{T} \end{array}\right]$$

where ${\left[\lambda_{ix_{j}}\right]}^{T}=\left[\begin{array}{ccc} \frac{\partial u_{i}}{\partial x_{j}} & \frac{\partial v_{i}}{\partial x_{j}} & \frac{\partial w_{i}}{\partial x_{j}} \end{array}\right]$ (j=1, 2, 3. For the hexahedral element of 20 nodes, i=1, 2, 3,…, 20).

(10) $$\left[H_{i}\right]={\left[\begin{array}{ccc} \left[\beta_{ix_{1}}\right] & 0 & 0\\ 0 & \left[\beta_{ix_{2}}\right] & 0\\ 0 & 0 & \left[\beta_{ix_{3}}\right] \end{array}\right]}^{T}$$

where $\left[\beta_{ix_{j}}\right]=\left[\begin{array}{ccc} \frac{\partial N_{i}}{\partial x_{j}} & \frac{\partial N_{i}}{\partial x_{j}} & \frac{\partial N_{i}}{\partial x_{j}} \end{array}\right]$, and $N_{i}$ is the element shape function [23].

Substituting Equations (6) and (7) into Equation (5) yields:

(11) $$\pi^{m}=\frac{1}{2}{\left[a\right]}^{T}\left[K^{m}\right]\left[a\right]-{\left[a\right]}^{T}\left[P\right]$$

where $\left[K^{m}\right]={\sum_{e}\left[G^{e}\right]}^{T}\left[K_{m}^{e}\right]\left[G^{e}\right]$, $\left[P\right]=\sum_{e}{\left[G^{e}\right]}^{T}\left[P^{e}\right]$, and $\left[a^{e}\right]=\left[G^{e}\right]\left[a\right]$ represent the nodal displacement column vector. $\left[G^{e}\right]$ is the transformation matrix; $\left[K_{m}^{e}\right]=\int_{V^{e}}{\left(\left[B_{L}\right]+\left[B_{NL}\right]\right)}^{T}\left[D\right]\left(\left[B_{L}\right]+\left[B_{NL}\right]\right)dv$ is the element stiffness matrix; and $\left[P^{e}\right]=\int_{V^{e}}{\left[N\right]}^{T}\left[f\right]dv+\int_{S^{e}}{\left[N\right]}^{T}\left[\bar{T}\right]dS$ represents the equivalent nodal load of the element.

Because $\delta \pi^{m}=0$,

(12) $$\left[K^{m}\right]\left[a\right]=\left[P\right]$$

Equation (12) is a nonlinear equation, where $\left[K^{m}\right]$ is a function of nodal displacement a. By introducing the displacement boundary conditions and solving the system of nonlinear equations by the iterative method, a nodal displacement column vector a can be obtained. Then, the volumetric strain at each point was obtained as follows:

(13) $$\theta =\frac{\Delta V}{V}=\varepsilon_{x}+\varepsilon_{y}+\varepsilon_{z}+\varepsilon_{x}\varepsilon_{y}+\varepsilon_{y}\varepsilon_{z}+\varepsilon_{x}\varepsilon_{z}+\varepsilon_{x}\varepsilon_{y}\varepsilon_{z}$$

As the elastic modulus of CNTs is much larger than that of polymers, the deformation of the composite mainly came from the polymer matrix. Therefore, the volume change of CNTs can be neglected. The volume fraction of CNTs can be represented as:

(14) $$f=\frac{V_{cnt}}{V}=\frac{V_{cnt}}{V_{0}+\Delta V}=\frac{V_{cnt}/V_{0}}{V_{0}/V_{0}+\Delta V/V_{0}}=\frac{f_{0}}{1+\theta }$$

where $V_{0}$ is the initial volume of the composite and $f_{0}$ is the initial volume fraction of the CNTs.

The average separation distance between adjacent CNTs was taken to be $d_{a}$. Several works have shown that $d_{a}$ is a function of the CNT volume fraction [24].

(15) $$d_{a}=d_{cp}{\left(f_{cp}/f\right)}^{1/3}$$

where $d_{cp}$ and $f_{cp}$ are the average separation distance and CNT volume fraction corresponding to the percolation threshold, respectively. It is shown that $d_{cp}=1.8\mathrm{nm}$ [13]. $f_{cp}$ can be approximated by the aspect ratio of the CNTs [25]:

(16) $$f_{cp}=\frac{9H(1-H)}{2+15H-9H^{2}}$$

where:

(17) $$H=\frac{1}{\alpha^{2}-1}\left[\frac{\alpha }{\sqrt{\alpha^{2}-1}}\ln\left(\alpha +\sqrt{\alpha^{2}-1}\right)-1\right]$$

$\alpha =L_{c}/D$. $L_{c}$ is the effective length of the CNT, and D is its diameter.

Physical studies have proven that there is a penetration effect between CNTs. Simmons derived the conductivity resulting from the perforation effect as [26]:

(18) $$\sigma_{m}=\frac{e^{2}{\left(2m\gamma \right)}^{1/2}}{h^{2}}\exp\left[-\frac{4\pi d_{a}}{h}{\left(2m\gamma \right)}^{1/2}\right]$$

where γ is the potential barrier height, representing the energy required for the electron transition through the matrix; m ($m=9.10938291\times 10^{-31}\;\mathrm{kg}$) is the electron mass; e ($e=1.602176565\times 10^{-19}\;C$) is the electric charge on an electron; and h ($h=6.626068\times 10^{-34}\;m^{2}\mathrm{kg}/s$) is the Planck constant.

The polymer matrix is usually an insulator, so the quantum tunneling conductivity between CNTs can be approximated to that of the matrix. Using the Mori–Tanaka method [27], the effective electrical conductivity of CNT composites $\sigma_{e}$ can be obtained as follows:

(19) $$\sigma_{e}=\sigma_{m}\left[1+\frac{f\cdot T}{1-f\left(1-T/\left(n-1\right)\right)}\right]$$

where

(20) $$T=\frac{n-1}{3}\left(\frac{2}{1+\left(n-1\right)S_{11}}+\frac{1}{1+\left(n-1\right)S_{33}}\right)$$

with

(21) $$S_{11}=\left\{\begin{array}{ll} \frac{\alpha }{2{\left(\alpha^{2}-1\right)}^{3/2}}\left[\alpha {\left(\alpha^{2}-1\right)}^{1/2}-\cosh^{-1}\alpha \right] & \alpha >1\\ \frac{\alpha }{2{\left(1-\alpha^{2}\right)}^{3/2}}\left[\cos^{-1}\alpha -\alpha {\left(1-\alpha^{2}\right)}^{1/2}\right] & \alpha <1 \end{array}\right.$$

where $S_{33}=1-2S_{11}$, $n=\sigma_{cnt}/\sigma_{m}$ and $\sigma_{cnt}$ is the conductivity of the CNTs.

Since the volume resistivity of CNT composites is ρ=1σe, the piezoresistivity of the CNT composites can be obtained by

(22) $$\frac{\Delta \rho }{\rho }=\frac{\rho -\rho_{0}}{\rho }=\frac{\rho }{\rho_{0}}-1=\frac{\sigma_{e0}}{\sigma_{e}}-1$$

where $\rho_{0}$ is the initial resistivity and $\sigma_{e0}$ is the initial conductivity.

Note that $\sigma_{e}$ is a function of composite volumetric strain, and thus, the piezoresistivity will vary with the deformation of the composites. However, for a structure, volume strain is not uniformly distributed; for example, in a beam, the lower part is characterized by expansion and the upper part by compression, so the application of Equation (22) in a practical structure is not convenient. Therefore, an alternative method for the calculation of piezoresistivity was developed as follows.

## 3. Piezoresistivity of CNT Composites Based on an Electric Field

**Figure 1:**
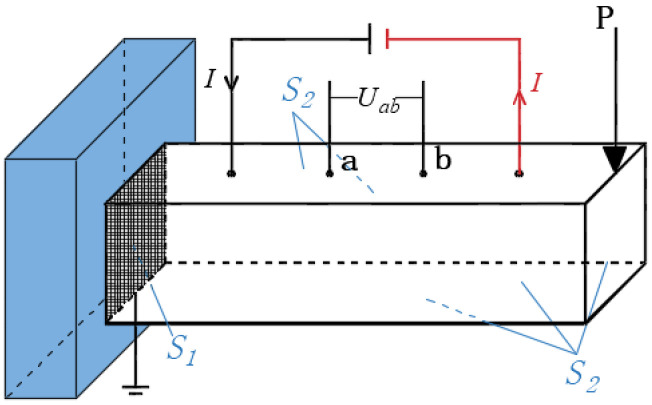
CNT composite under an electric field.

Let the CNT composite be subjected to an electric field, as shown in Figure 1. The electric potential φ, current density J tensor, and electric field strength E tensor of each point inside the composite satisfy the following relationships:

(23) E=−∇φ

(24) $$J=\sigma_{e}\cdot E$$

(25) ∇⋅J=0

Boundary conditions:

(26) $$\mathrm{Boundary}\;S_{1}:\;\varphi =\bar{\varphi }$$

(27) $$\mathrm{Boundary}\;S_{2}:\;J\cdot n-\bar{q}=0$$

where $\bar{q}$ and $\bar{\varphi }$ are the surface current density and electric potentials on the boundary surface, respectively. $\sigma_{e}$ is the conductivity tensor, and for isotropic case, $\sigma_{e}=\sigma_{e}\delta$, where δ is the unit tensor.

The equivalent integral form of Equation (25) and boundary conditions (27) is:

(28) $$\int_{V}v\left(\nabla \cdot J\right)dV+\int_{S_{2}}\bar{v}\left(J\cdot n-\bar{q}\right)dS=0$$

where v and $\bar{v}$ are the weight function. The variation in electric potential was selected as the weight function, i.e., v(x,y,z)=δφ(x,y,z), and $\bar{v}=-v$ was adopted. Considering (23) and (24), Equation (28) was rewritten as

(29) δπ=0

where

(30) $$\pi =\int_{V}\frac{1}{2}\left(\nabla \varphi \right)\cdot \sigma_{e}\cdot \left(\nabla \varphi \right)dV-\int_{S_{2}}-\varphi \bar{q}dS$$

A 20-node hexahedron iso-parametric element was used for finite element analysis. The computational domain was divided into a number of hexahedral elements, and both the corner points and midpoints of each side were taken as nodes. For a particular element (e), the electric potential could be determined by:

(31) $$\varphi^{e}(x,y,z)=\left[N\right]\left[\varphi^{e}\right]$$

where

(32) $$\left[N\right]=\left[N_{1},N_{2},\ldots ,N_{20}\right],\left[\varphi^{e}\right]={\left[\varphi_{1}^{e},\varphi_{2}^{e}\ldots ,\varphi_{20}^{e}\right]}^{T}$$

where $N_{i}$ is the shape function and $\varphi_{i}^{e}$ is the nodal electric potential.

(33) $$\nabla \varphi^{e}=\left[B\right]\left[\varphi^{e}\right]$$

where

(34) $$\left[B\right]=\left[\begin{array}{cccc} \frac{\partial N_{1}}{\partial x} & \frac{\partial N_{2}}{\partial x} & \ldots & \frac{\partial N_{20}}{\partial x}\\ \frac{\partial N_{1}}{\partial y} & \frac{\partial N_{2}}{\partial y} & \ldots & \frac{\partial N_{20}}{\partial y}\\ \frac{\partial N_{1}}{\partial z} & \frac{\partial N_{2}}{\partial z} & \ldots & \frac{\partial N_{20}}{\partial z} \end{array}\right]$$

After discretization and substituting Equations (33) and (31) into Equation (30), we obtained:

(35) $$\pi =\sum_{e}\left\{\frac{1}{2}{\left[\varphi^{e}\right]}^{T}\left[K^{e}\right]\left[\varphi^{e}\right]\right\}-\sum_{e}{\left[\varphi^{e}\right]}^{T}\left[q^{e}\right]$$

where $\left[K^{e}\right]=\int_{V^{e}}{\left[B\right]}^{T}\left[\sigma^{e}\right]\left[B\right]dV$, and $\left[q^{e}\right]=\int_{S_{2}^{e}}-{\left[N\right]}^{T}\left[\bar{q}\right]dS$.

The nodal electric potential vector of each element $\left[\varphi^{e}\right]$ can be written as:

(36) $$\left[\varphi^{e}\right]=\left[G^{e}\right]\left[\varphi \right],\;\left[\varphi \right]=\left[\varphi_{1},\varphi_{2},\ldots ,\varphi_{20}\right]$$

where $\left[G^{e}\right]$ is the transformation matrix, $\left[\varphi \right]$ denotes the electric potential of all nodes, and $\varphi_{k}\left(k=1,\;2,\;3\ldots ,\;20\right)$ denotes a particular nodal electric potential. Then, Equation (35) becomes:

(37) $$\pi =\frac{1}{2}{\left[\varphi \right]}^{T}\left[K\right]\left[\varphi \right]-{\left[\varphi \right]}^{T}\left[q\right]$$

where $\left[K\right]=\sum_{e}\left({\left[G^{e}\right]}^{T}\left[K^{e}\right]\left[G^{e}\right]\right)$, and $\left[q\right]=\sum_{e}\left({\left[G^{e}\right]}^{T}\left[q^{e}\right]\right)$.

Substitution of Equation (37) into Equation (29) yields

(38) $$\left[K\right]\left[\varphi \right]=\left[q\right]$$

Equation (38) is a set of linear equations. In order to solve Equation (38), boundary conditions (26) must be considered.

The electric potential of all nodes can be obtained by solving Equation (38). The voltage between point a and b is:

(39) $$U_{ab}=\varphi_{a}-\varphi_{b}$$

The volume resistivity, voltage *U*, and current *I* satisfy the following relationships:

(40) $$\rho =C\frac{U}{I}$$

where C is a constant and depends only on the geometry.

The piezoresistivity of CNT composites can be determined by a coupling analysis of both the mechanical and electric fields. As shown in Figure 1, for each step of mechanical loading, the finite element analysis as stated in Section 2 was carried out to obtain the elastic deformation. Then, both the volume strain θ and electrical conductivity $\sigma_{e}$ of each point were calculated according to Equations (13)–(19). Thereafter, the voltage between two points before and after loading with the same current I was obtained with the electric field. Therefore, from Equation (40), the piezoresistivity of CNT composites can be represented as:

(41) $$\frac{\Delta \rho }{\rho }=\frac{U_{ab}-U_{ab}^{0}}{U_{ab}^{0}}$$

where $U_{ab}^{0}$ is the initial voltage before loading between a and b, and $U_{ab}$ is the voltage after loading. Using the above coupling analysis, piezoresistivity can be determined for any position in a non-uniformly deformed structure.

It should be noted that Equation (41) is very important for practical applications. With this equation, we can simply measure the piezoresistivity of CNT composites by measuring the voltage before and after loading.

## 4. Method Validation

**Figure 2:**
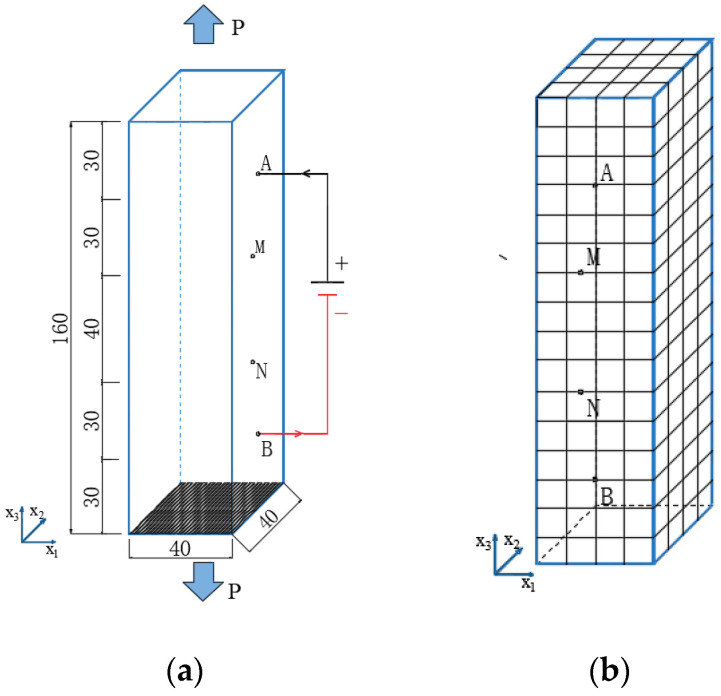
Testing specimen (**a**) and finite element mesh (**b**) for the piezoresistivity of CNT composites.

**Figure 3:**
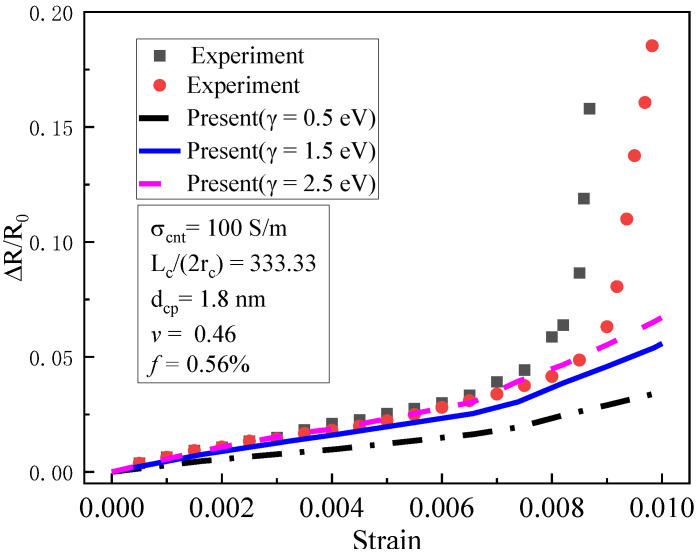
Piezoresistivity of CNT composites: experimental data and prediction ( *f*_0_ = 0.56%) [19].

**Figure 4:**
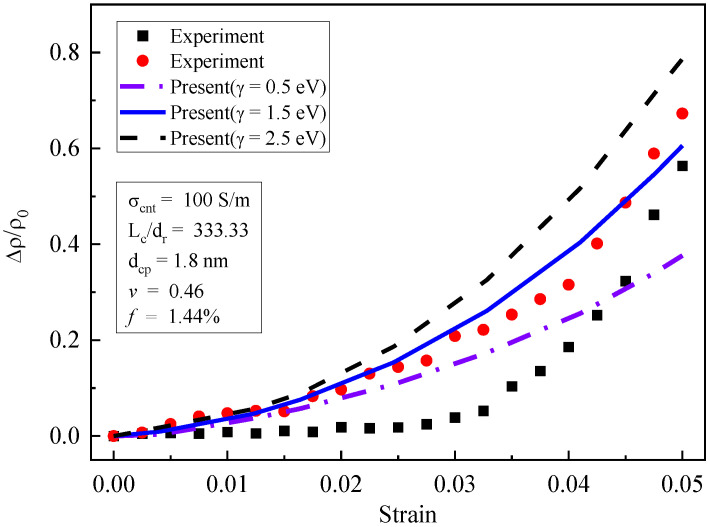
Piezoresistivity of CNT composites: experimental data and prediction (*f*_0_ = 1.44%) [19].

The piezoresistivity of multi-walled CNT composites under tension was studied in reference [19]. The matrix material was PEO (polyethylene oxide), and the initial volume fractions of multi-walled CNTs were 0.56% and 1.44%, respectively. The basic physical parameters of the composite were as follows: a Poisson’s ratio of 0.46 [19] and an elastic modulus of 7.86 MPA [19]. The CNTs’ average diameter D was 15 nm [11]; the average length $L_{c}$ was 5 μm [11], and the conductivity of the carbon nanotubes was 100 S/m [11] $d_{cp}=1.8\;\mathrm{nm}$. The barrier height of the polymers was usually 0.5–2.5 eV [7]. Therefore, 0.5, 1.5, and 2.5 eV were adopted. The experimental specimen (a) and the finite element mesh (b) for the piezoresistivity of CNT composites are shown in Figure 2. The dimension of the specimens was 160 mm×40 mm×40 mm. The CNTs were initially uniformly distributed in a resin matrix. The external electric field was applied at point A and B, and the ends of the specimen were subjected to tensile loads. The iso-parametric element of hexahedron 20 nodes was used in this simulation. The domain was divided into 16×4×4=256 elements, in which there were a total of 1505 nodes. The piezoresistivity obtained according to Equation (41) was compared with the experimental results shown in Figure 3 and Figure 4, respectively.

As shown in Figure 3, the piezoresistivity of $f_{0}$ = 0.56% was compared with the experimental results. When the strain increased from 0 to 0.6%, both the test results and the simulation results increased linearly, and geometric nonlinearity was negligible. We noted that when the barrier height was between 1.5 and 2.5 eV, the simulation results agreed well with the test results. When the strain was between 0.6% and 0.8%, the simulation results presented a similar non-linear growth trend as the test results, which was caused by the geometric nonlinearity of the specimens. However, when the strain was greater than 0.008, the piezoresistivity increased sharply and tended towards infinity, indicating that damages or fractures in the matrix probably occurred between CNTs. Although the simulation results also show a very large non-linear trend, there was still a significant difference between the simulation and the tests, since the present approach did not incorporate the effect of damages or fractures in the composites.

Figure 4 shows the results of piezoresistivity for *f*_0_ = 1.44%. Under the same experimental conditions, highly discrete results were obtained from two specimens, which may have been due to the nonuniform distribution of the CNTs. When the strain increased from 0 to 1%, both the test results and the simulation results increased linearly, and the geometric nonlinearity had little influence. When the strain was greater than 1%, geometric nonlinearity played an important role, leading to the non-linear growth of piezoresistivity. It can be observed that the simulation results reflected this growth trend well. With the very large strain of 5%, the simulation results were in good agreement with the experimental results.

The above analyses show that, although further studies are still needed, the present approach can provide a reasonable prediction of the piezoresistivity of CNT composites.

## 5. Analysis of Parameters Affecting Piezoresistivity

Theoretically, the parameters which may affect the conductivity of CNT composites include both the aspect ratio and the conductivity of CNTs, the potential barrier height of the matrix, and the initial average separation distance between adjacent CNTs. In the following section, their effects on the prediction of piezoresistivity are analyzed according to Equation (41).

### 5.1. Influence of Aspect Ratio α

**Figure 5:**
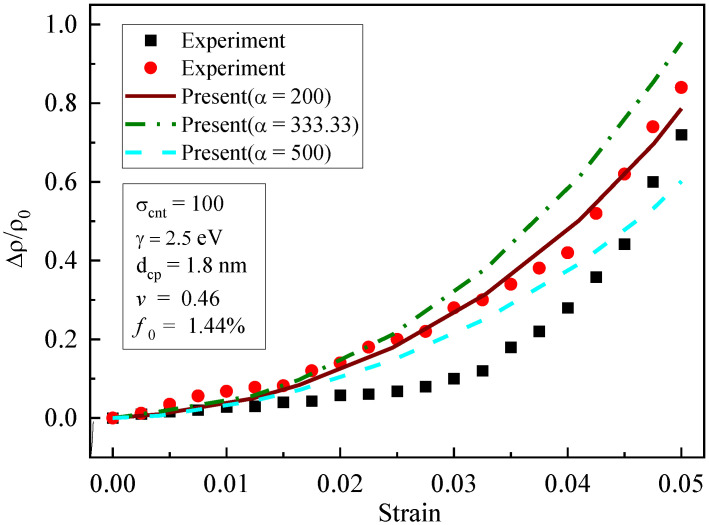
Influence of aspect ratio on piezoresistivity [19].

The effects of the aspect ratio (α=200,333.33, and 500) on the prediction of piezoresistivity are illustrated in Figure 5. It is shown that the influence of α was weak when the strain was low, and the effect of α became more remarkable with the increase in strain. With the increase in the aspect ratio, the piezoresistivity of the composites decreased gradually. This is because the aspect ratio affected the formation of the conductive network and the quantum tunneling effect, and hence affected the conductivity of the composites. Therefore, when the strain was large, the influence of the aspect ratio on the piezoresistivity of the composites was remarkable.

### 5.2. Influence of Conductivity of CNTs

**Figure 6:**
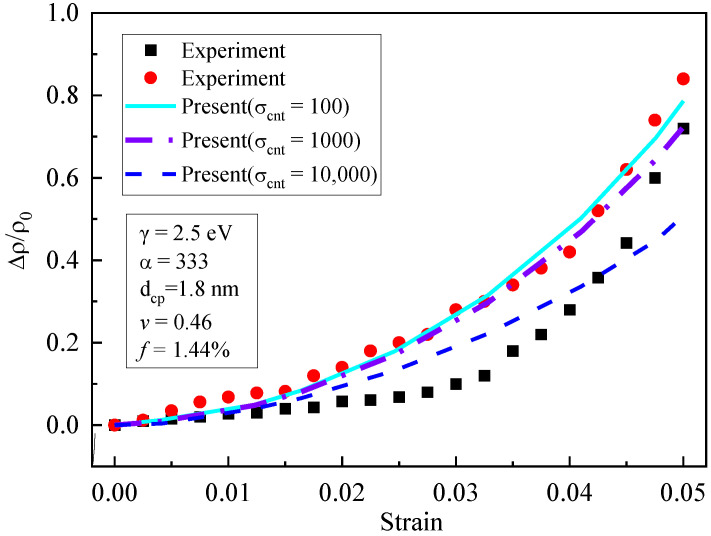
Influence of CNT conductivity on piezoresistivity [19].

As shown in Figure 6, the influence of the conductivity of the CNTs ($\sigma_{cnT}=100\;S/m$, 1000 S/m and 10,000 S/m) on the piezoresistivity was analyzed. The figure shows that the influence of the conductivity of the CNTs on piezoresistivity was not obvious when the strain is small; when the strain increased, the piezoresistivity decreased with the increase in the CNTs’ conductivity. Particularly, when $\sigma_{cnt}=$ 10,000, the decreasing trend became more remarkable. This is because the significant CNTs conductivity led to insensitivity of the composite’s conductivity to strain, which weakened the composite’s piezoresistivity.

### 5.3. Influence of Potential Barrier Height

**Figure 7:**
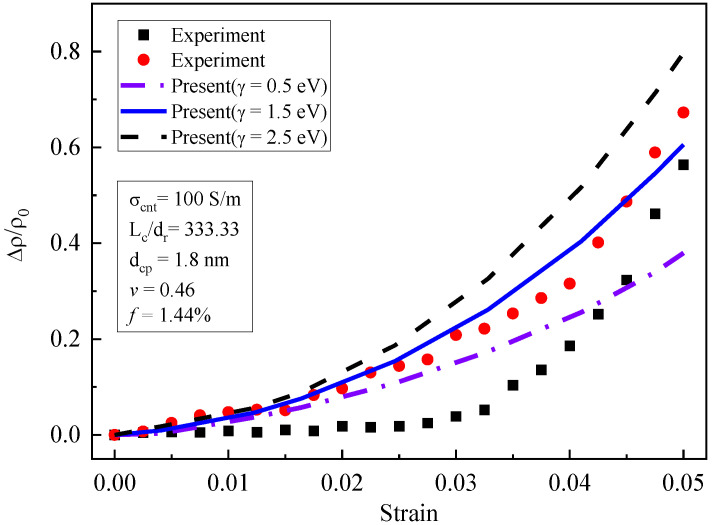
Influence of potential barrier height on piezoresistivity [19].

Shown in Figure 7 are the piezoresistivity curves for different barrier heights (γ=0.5 eV, 1.5 eV, and 2.5 eV). When the potential barrier height increased from 0.5 eV to 1.5 eV, the piezoresistivity increased obviously. However, the increase amplitude decreased when the barrier height increased from 1.5 eV to 2.5 eV. The potential barrier height represents the energy required for the electron to transition through the matrix. High potential barrier height values made electron tunneling more difficult, which significantly affected the quantum tunneling conductivity. Quantum tunneling conductivity was more sensitive to composite deformation than CNT conductivity. Therefore, piezoresistivity was sensitive to changes in the potential barrier height.

### 5.4. Influence of Initial Average Separation Distance $d_{cp}$

**Figure 8:**
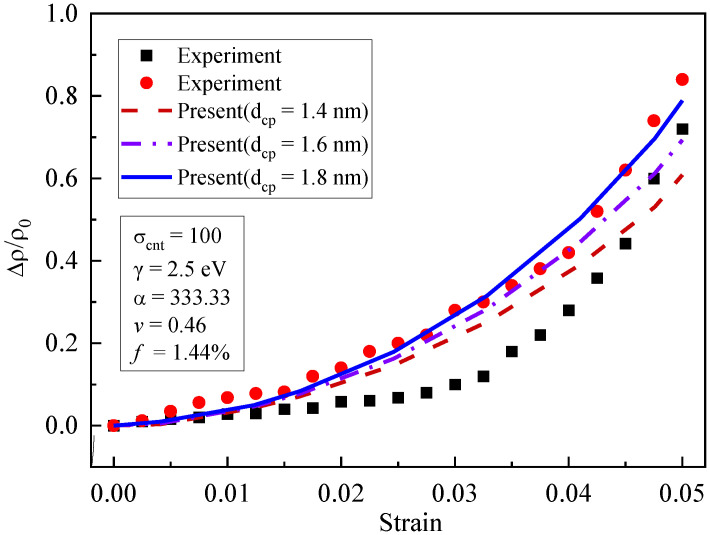
Influence of initial average separation distance on piezoresistivity [19].

Shown in Figure 8 are the curves of piezoresistivity for different initial average separation distances ($d_{cp}=1.4\;\mathrm{nm}$, 1.6 nm, and 1.8 nm). The influence of the initial average separation distance on the piezoresistivity was obvious. When $d_{cp}$ increased from 1.4 nm to 1.8 nm, the piezoresistivity increased. The increasing trend became more obvious when the strain grew larger. The initial average separation distance was related to the quantum tunneling conductivity, which was sensitive to composite deformation. Large values of $d_{cp}$ made quantum tunneling more difficult, which increased the resistivity of the composites. Therefore, after the deformation of the composites, the piezoresistivity for large $d_{cp}$ values was higher than that for small $d_{cp}$ values.

## 6. Application

**Figure 9:**
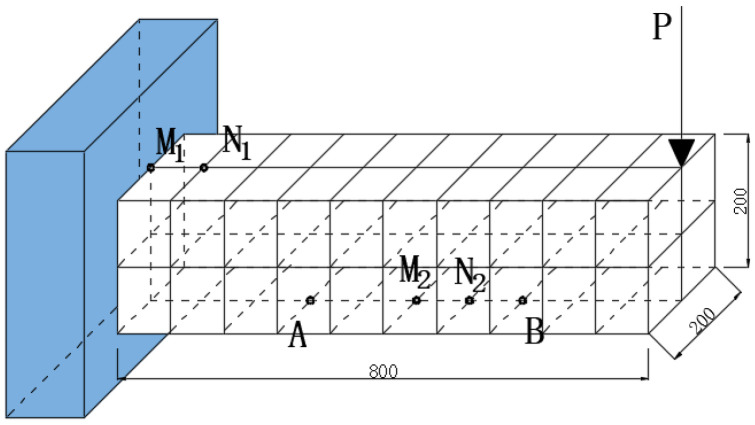
Finite element mesh and measurement points.

In order to demonstrate the application of the present approach to engineering structures, a finite element analysis was conducted on the cantilever beam shown in Figure 1. The dimensions of beam were 800 mm × 200 mm × 200 mm, and there was a concentrated force P acting on its cantilever end. The finite element mesh is shown in Figure 9. The beam was composed of CNT composites, with an elastic modulus of E=3 GPa and a Poisson’s ratio of v=0.3. The initial CNT volume fraction was $f_{0}=1.21\%$; the average diameter of the CNTs = 15 nm; and the average length $L_{c}$ = 5 μm. The CNT conductivity was 10,000 S/m, γ=0.5 eV, and $d_{cp}=1.8\;\mathrm{nm}$.

A steady-state electric field was applied before loading, with the current flowing in at point A at the bottom and flowing out at point B. Two sets of measuring points, $M_{1}$$N_{1}$ and $M_{2}$$N_{2}$, were used to test the voltage changes in the tension and compression zones, respectively, in order to determine the corresponding piezoresistive effect.

**Figure 10:**
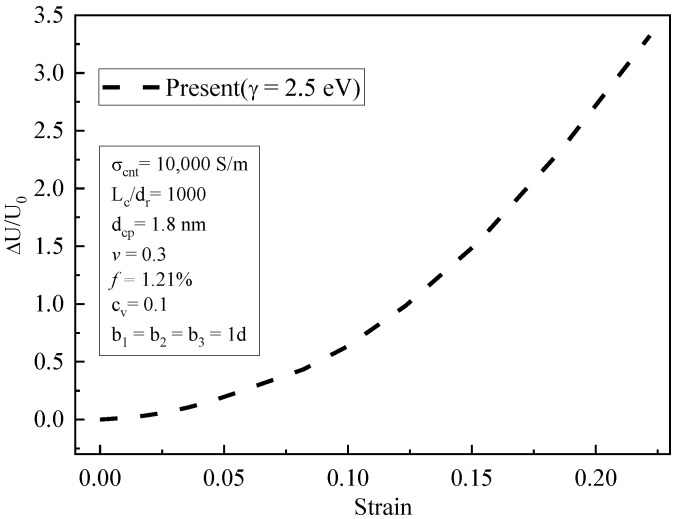
The relative variation in voltage with tensile strain (simulation results).

Figure 10 shows the variation curve of relative voltage in the tensile zone with the variation in the strain. Under concentrated force, the strain increased with the loads, and the piezoresistive behavior of CNT composites increased significantly. When the strain was low, the relative change in voltage increased linearly. With the further increase in strain, the piezoresistive behavior gradually became non-linear. The reason for this is that when the tensile strain was high, it led to an increase in the spacing between CNTs. The tunneling conductivity changed exponentially due to the influence of geometric nonlinearity, resulting in a non-linear increase in the relative voltage variation at the macro level. When the strain was small, the nonlinear term of volume strain (Equation (13)) was so small that it could be ignored, and therefore, the piezoresistive behavior was linear.

**Figure 11:**
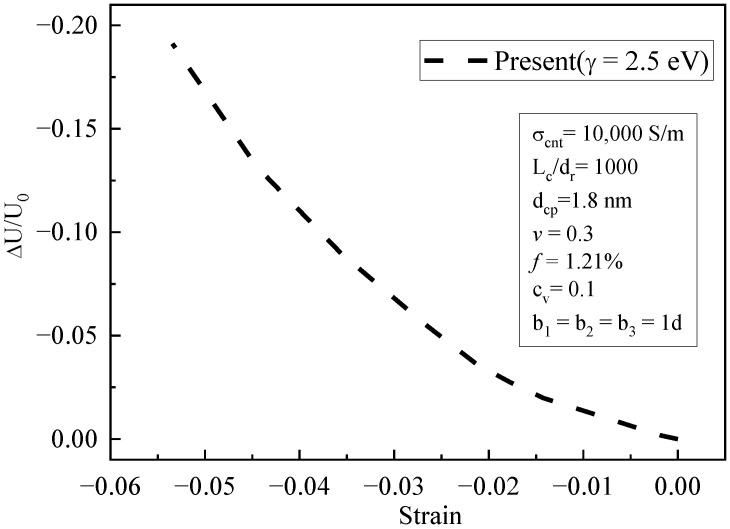
The relative variation in voltage with compressive strain (simulation results).

Figure 11 shows that the piezoresistive behavior in the compressive zone decreased with increasing compressive strain. When the compressive strain was low, its linear trend was more obvious. As the compressive strain increased, the relative change in voltage gradually became non-linear. Compared with the tensile zone, this trend was smaller, and the variation in piezoresistive behavior was weaker. This is because the tensile strain was greater than the compressive strain under the same load. Therefore, the piezoresistive behavior due to geometric nonlinearity in the compressive zone was weaker.

## 7. Conclusions

(1) Based on the Mori–Tanaka method and considering the quantum tunneling effect between CNTs, an approach was proposed by which to calculate the equivalent electrical conductivity of CNT polymer composites.
(2) A three-dimensional theory was established for the piezoresistivity of CNT composites which incorporated the effect of the composites’ geometric nonlinearity. The theory is dependent only on some basic physical parameters of the materials. The finite element formula of the theory for the numerical calculation of piezoresistivity was presented from the analysis of both elastic and electric fields. The simulation results were in good agreement with the experimental data.
(3) Both the aspect ratio and the conductivity of the CNTs, as well as the initial average separation distance between CNTs and the barrier height of the polymers, may lead to a change in composite conductivity, which, in turn, would affect the piezoresistivity of composites.

The theory and numerical approach are expected to be applied to complex structural forms, including rods, plates, and shells and planar and solid structures, enabling them to have self-monitoring capabilities. By measuring the changes in their own electrical properties, real-time evaluation of structural deformation may be achieved. To achieve this goal, further research is still needed in the future.

## Data Availability

All the data used in this paper in provided in the manuscript.
